# Storage Capacity of Entomopathogenic Nematodes in Barricade^®^ Gel and Potassium Polyacrylate Hydrogel

**DOI:** 10.2478/jofnem-2025-0014

**Published:** 2025-06-21

**Authors:** Sinethemba Zulu, Tshimangadzo Ramakuwela, Hugues Baimey, Mark Laing, David Shapiro-Ilan, Nicolene Cochrane

**Affiliations:** ARC-Small Grains, Lindley Road R76, Bethlehem, 9701 South Africa; Infruitec-Nietvoorbij, Helshoogte Rd, Stellenbosch Central, Stellenbosch, 7599, South Africa; Department of Plant and Soil Sciences, University of Pretoria, Hatfield, 0084, South Africa; Department of Crop Production, University of Parakou, Parakou, Benin; College of Agriculture, Engineering and Science, University of KwaZulu-Natal/African Centre for Crop Improvement, Scottsville, 3209, South Africa; USDA-ARS, SAA, 21 Dunbar Road, Byron, GA 31008, United State of America; Agrimetrics, (CO), Agricultural Research Council Central, Parkstreet, 1134 Hatfield, Pretoria, 0083, South Africa

**Keywords:** Biological control, formulation technology, Heterorhabditidae, *in vivo* and *in vitro* production, shelf life, Steinernematidae

## Abstract

Entomopathogenic nematodes (EPNs) used as biocontrol agents are sensitive to ultraviolet (UV) light, high temperature, and desiccation. Thus, formulations have been developed to protect EPNs during application. However, the ability of these formulations to enhance storage capacity has not been investigated. This study analyzed storage capacity (survival and efficacy) of EPN species, *Heterorhabditis bacteriophora* (SGI 245), *Steinernema tophus* (ROOI 352) and *Steinernema innovationi* (SG I35) produced either *in vivo* or *in vitro* and formulated in 2% gel of either Barricade^®^ or potassium polyacrylate hydrogel (PPH). The formulations were stored at 10°C and survival of the infective juvenile nematodes (IJs) was evaluated at two-weeks intervals for eight weeks. The efficacy of formulated nematodes was evaluated using *Tenebrio molitor*. After two weeks, all control treatments had 0% IJ survival for all the three isolates, whereas the gel formulations exhibited 58–76% survival. The three isolates in both the Barricade^®^ gel and PPH formulations exhibited 37–69% IJ survival at six weeks, which declined to 0–13% after eight weeks. Both formulations of the three isolates were 60–90% effective at six weeks. The *in vitro*-produced IJs had a higher survival than the *in vivo*-produced IJs for *S. innovationi* and *H. bacteriophora*. However, the *in vivo*-produced IJs were more effective at killing *T. molitor* than the *in vitro*-produced EPNs for *H. bacteriophora* but not the *Steinernema* spp. In conclusion, Barricade^®^ and PPH gel formulations substantially increased survival of the three EPN species during storage.

Entomopathogenic nematodes (EPNs) are roundworms that are obligate parasites of insects in nature (Shapiro-Ilan and Lewis, 2024). EPNs are found worldwide in the soil except in Antarctica (Nouh, 2021). Isolates of two families, Steinernematidae and Heterorhabditidae, are of interest as biocontrol agents because of their ability to attack and kill a wide range of insects within 48 hours. Insect mortality is achieved with the aid of a mutualistic interaction between the EPNs and symbiotic intestinal bacteria of genera *Xenorhabdus* and *Photorhabdus* of Steinernematidae and Heterorhabditidae, respectively. The non-feeding infective juveniles (IJs) have the ability to search for and infect hosts. Infection occurs when IJs gain entrance into the insect hemocoel through the natural openings of the host and through the cuticle of some species. Inside the target host insect, symbiotic bacteria are then released into the hemocoel, where the bacteria reproduce and release pathogenic factors such as secondary metabolites, toxins, hydrolytic enzymes, hemolysins, and antimicrobial compounds, which contribute to killing the target insect (Yooyangket et al., 2018; Tarasco et al., 2023). The bacteria create a suitable and conducive environment for reproduction and multiplication of nematodes. This leads to the quick death of the host. Upon nutrient depletion, the next generation of IJs exit the host cadaver, carrying the symbiotic bacteria in their intestines, in search of new hosts (Vashisth et al., 2013).

Although EPNs have been proven effective in controlling insects, they are fragile organisms, and there are some environmental conditions that negatively affect their survival, reproduction, and efficacy. These includes high temperature, UV light, and desiccation (Acar et al., 2022). Therefore, there is a need to develop nematode formulations that enable the EPNs to tolerate a range of adverse environmental conditions. Acar et al. (2022) evaluated a number of products that could potentially enhance the survival of EPNs such as chemicals that block or absorb UV radiation, and which slow desiccation, including P-amino benzoic acid (PABA), octyl methoxycinnamate (OMC), Congo red, titanium dioxide, and zinc. PABA and OMC are widely used in human sunscreens (Acar et al., 2022). Despite these chemicals showing potential, other alternatives are needed that are cost-effective and environmentally friendly. These may include the use of Barricade^®^ gel (Shapiro-Ilan et al., 2010; 2016) and hydrogels such as potassium polyacrylate hydrogel (PPH). Potassium polyacrylate hydrogel (PPH) has not been included in EPN studies previously: this is a novel formulation. Barricade^®^ gel is a trademarked product made from absorbent polymers and is largely sold as a fire protectant. Barricade^®^ gel has shown potential in protecting nematodes from desiccation and UV light (Shapiro-Ilan et al., 2010; 2016), and thereby improving EPNs efficacy and longevity. It is nontoxic to the environment and easy to apply (Shapiro-Ilan and Goolsby, 2021).

Superabsorbent hydrogels have been widely utilized in the agricultural sector for over 40 years. Their main use is to enhance water availability for plants by increasing the water-holding properties of the soil, especially sandy soils; reducing irrigation frequencies; and minimizing compaction, soil salinization, and water run-off (Dhiman et al., 2020). Potassium polyacrylate is considered environmentally friendly and has the ability to save water, fertilizer, and labor; moreover, potassium polyacrylate improves nutrient usage and soil conditions for plants (Dhiman et al., 2020). Although previous studies have explored different gel formulations (Andalo et al., 2010; Leite et al., 2018) as protective mechanisms for EPNs during field application (especially aboveground applications), the potential of these formulations to enhance EPNs storage capacity (i.e., Barricade^®^ and PPH formulations) has not been investigated. The aim of this study was to assess the use of Barricade^®^ gel and PPH formulations on the storage capacity (survival and efficacy) of EPNs under laboratory conditions. This is the first study to investigate PPH formulation, which was selected because of its advantages mentioned above.

## Materials and Methods

### Source of EPN isolates

The three EPN isolates used, *Heterorhabditis bacteriophora* (Rhabditida: Heterorhabditidae) Poinar 1976 (SGI 245), *Steinernema tophus* (Nematoda: Steinernematidae) Çimen 2014 (ROOI 352) and *Steinernema innovationi* (Panagrolaimomorpha: Steinernematidae) Çimen, Lee, Hatting and Stock 2014 (SGI 35), were originally isolated from Fouriesburg in the Free State province of South Africa (28° 31′ 59.7″S 28° 10′ 31.), and the former isolated from a rooibos tea soil sample in Clanwilliam, Western Cape Province, South Africa (32°10′43″S 18°53′28″E), respectively (Hatting et al., 2009). Isolates were revived by passaging through *Galleria mellonella* L. (Lepidoptera: Pyralidae), as per Kaya and Stock (1997). The number of IJs were adjusted to 1000 IJs ml^–1^. One ml of IJs suspension was distributed evenly on a 90 mm filter paper in a lid of a 100 mm Petri dish. Ten larvae of *G. mellonella* were added in the Petri dish, covered with a lid and stored in a ziplock plastic bag. The Petri dishes were then incubated at 25°C for three days. Cadavers were placed on White traps (Hazir et al., 2022). After 8 to 14 days, IJs were harvested and washed three times (Kaya and Stock, 1997). The collected IJs were then stored in sterile distilled water in horizontal culture flasks at 10°C and were used within two weeks after harvesting for *in vivo* and *in vitro* mass production.

### *In vivo* and *in vitro* IJs production

Two EPNs production methods were used, *in vivo* and *in vitro*, in order to compare the efficacy and survival of the new *in vitro* method developed by Ramakuwela et al. (2016) and the widely used *in vivo* method. Entomopathogenic nematodes were produced *in vivo* using the protocol described by Ramakuwela et al. (2018) and were then used immediately to create the two gel formulations.

The *in vitro* production of EPNs was achieved by means of a medium, based on pureed larvae of *Musca domestica* Linnaeus, 1758 (Diptera: Muscidae) according to Ramakuwela et al. (2016). *Musca domestica* larvae were reared at the Agricultural Research Council – Small Grains (ARC-SG) Insect Pathology research laboratory on a diet containing 2 kg bran, 300 g Nespray^®^ milk powder (Nestle^®^ Nespray^®^ FortiGrow™), 6 g sodium benzoate, 20 g brewer’s yeast, and 3 L lukewarm water. Five grams of *M. domestica* larvae were pureed using a blender, 0.15 g canola oil was added, and the mixture was absorbed in sponge cubes. The media were then placed inside 250 ml conical flasks, closed with cotton wool, covered with a foil and an elastic band to secure the foil, before autoclaving at 121°C for 15 min. The media was allowed to cool down and was then inoculated with symbiotic bacteria and IJs at a concentration of 100 IJs ml^–1^ and incubated at 25°C for four weeks (Ramakuwela et al., 2018).

### EPNs survival in different formulations and subsequent infectivity

Both *in vivo* and *in vitro*-produced IJs were washed with sterile distilled water three times, to eliminate dead IJs by allowing the live IJs to settle at the bottom of a 50 ml centrifuge tubes. Barricade^®^ gel (AECI Specialty Chemicals) and PPH (Qingdao SOCO Material CO., Ltd) formulations at 2% were prepared using IJs of each EPN species and each production method by mixing 2 ml of Barricade^®^ gel or PPH and 98 ml water containing 100 000 IJs ml^–1^ in a 150 ml glass beaker. The high concentration of IJs (see Hazir et al. 2022) was used because that is more reflective of commercial storage conditions than short-term storage for normal laboratory experiments. Thereafter, 2 ml samples of the formulations were transferred into 2 ml Eppendorf tubes placed on a rack and covered with foil. The formulations were incubated at 10 °C. Eighteen replicates were prepared per formulation and three replicates were analyzed using destructive sampling at two-weeks intervals. At each sampling time, formulations were re-suspended in 20 ml of distilled water in a Petri dish. The survival of the formulated IJs was assessed by loading the diluted suspension onto a nematode counting slide (ThermoFisher Scientific) and counting 50 IJs as live or dead under a stereomicroscope per sample.

The IJs from the counting dishes were then adjusted to a concentration of 1000 live IJs ml^–1^ and ten larvae of *Tenebrio molitor* Linnaeus, 1758 (Coleoptera: Tenebrionidae) were infected with 1 ml of IJ suspension in a 90 mm Petri dish lined with a filter paper to assess the infectivity of the surviving nematodes of each of the formulations. Plates were then incubated at 25 °C for 48 hrs. The control treatments were prepared using only the two EPNs suspensions formulated in sterile distilled water without the gels. Mortality of mealworms was assessed after 48 hrs. The experiments were repeated three times using different nematode batches at different times.

In summary, there were three EPN isolates x two methods of production (*in vivo* and *in vitro*) x three formulations (control, Barricade^®^ gel, and PPH), creating a total of 18 discrete EPN products tested.

### Statistical analysis

Collected data was analyzed using the analysis of variance (ANOVA) on Genstat or SAS statistical software and the GLM Procedure (Fisher, 1970). The area under the disease progress curve (AUDPC) was also utilized to compare IJ survival and efficacy against *T. molitor* across different formulations (Jeger et al., 2001).

## Results

On Day 1, the survival of the EPNs of all three isolates, *in vivo* and *in vitro*-produced, both formulations, and the controls were 100% viable (Table 1). There was significant difference in the survival of the three EPNs in either formulation compared with the controls. Control treatments survival was 0% after two weeks, whereas the survival of the *in vivo* EPNs in PPH was 59% for *H. bacteriophora* SGI 245, 76% for *S. tophus* and 68% for *S. innovationi*. The survival of EPNs continued to decrease to <15% at eight weeks, with the survival of *in vivo*-grown EPNs in PPH at 13% for *H. bacteriophora* SGI 245, 4% for *S. tophus* and 0% for *S. innovationi*. The survival of *in vitro*-produced EPNs in PPH at eight weeks was 10% for *H. bacteriophora* SGI 245, 2% for *S. tophus* and 1% for *S. innovationi*. The formulations were statistically analyzed and compared per two-weeks intervals. The top performing groups were significant letters “a” and “ab”, the worst performing category was significant letter “c” (Table 1).

**Table 1:** Survival of entomopathogenic nematodes produced *in vivo* and *in vitro* and formulated in either Barricade^®^ gel (B) or Potassium Polyacrylate Hydrogel (PPH) (LSD (*P* < 0001) = 20.64).

**Strain**	**Formulation**	**Day 1**	**2 weeks**	**4 weeks**	**6 weeks**	**8 weeks**
**SGI 245** *Heterorhabditis bacteriophora*	*In vivo* B	100 a	59 a	56 a	37 b	13 a
	*In vitro* B	100 a	64 a	65 a	67 a	8 a
	*In vivo* PPH	100 a	59 a	55 a	37 b	13 a
	*In vitro* PPH	100 a	61 a	63 a	68 a	10 a
	*In vivo* control	100 a	0 b	^*^	^*^	^*^
	*In vitro* control	100 a	0 b	^*^	^*^	^*^
**ROOI 352** *Steinernema tophus*	*In vivo* B	100 a	76 a	60 a	47 b	4 a
	*In vitro* B	100 a	62 b	57 a	66 a	^*^
	*In vivo* PPH	100 a	76 a	60 a	47 b	4 a
	*In vitro* PPH	100 a	62 b	63 a	66 c	2 a
	*In vivo* control	100 a	0 c	^*^	^*^	^*^
	*In vitro* control	100 a	0 c	^*^	^*^	^*^
**SGI 35** *Steinernema innovationi*	*In vivo* B	100 a	68 a	53 a	47 b	^*^
	*In vitro* B	100 a	58 b	63 a	68 a	^*^
	*In vivo* PPH	100 a	68 a	53 a	47 b	^*^
	*In vitro* PPH	100 a	60 b	69 a	69 a	1
	*In vivo* control	100 a	0 c	^*^	^*^	^*^
	*In vitro* control	100 a	0 c	^*^	^*^	^*^

* no infective juvenile survival.

**Figure 1:**
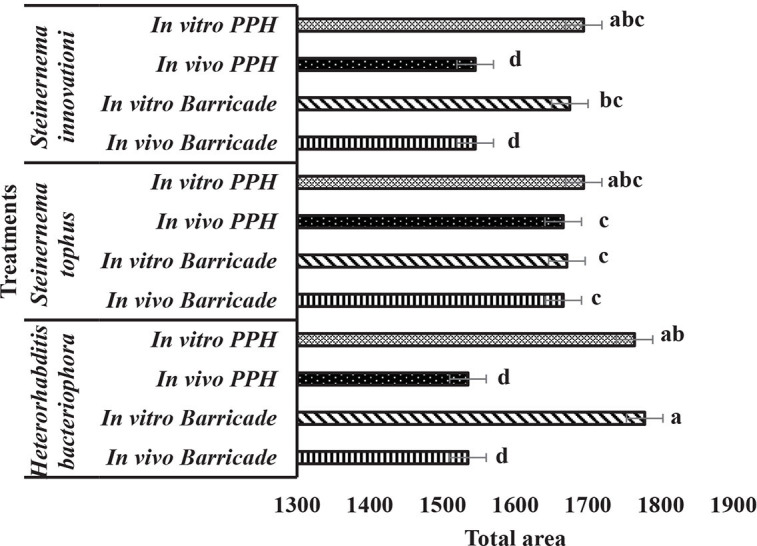
Total area under the curve analyzed using area under the disease progress curve (AUDPC) statistical analyses for the survival of three entomopathogenic nematode species, produced in vivo or in vitro and formulated in Barricade^®^ gel (B) or Potassium Polyacrylate Hydrogel (PPH), assessed at two weeks intervals over a period of eight weeks. Different letters at each bar indicate significant differences between treatments (α = 0.05), compared for each nematode species separately.

Figure 1 shows the total area under the survival curve. *In vivo*-produced EPNs in Barricade^®^ gel were the lowest, 1535 for *H. bacteriophora* and 1666, for *S. tophus* and 1545 for *S. innovationi*. The *in vitro*-produced EPNs in Barricade^®^ gel had areas under the survival curve of 1778 for *H. bacteriophora* as the highest. For the PPH formulations of *in vivo*-produced EPNs, the areas under the survival curve of 1666 for *S. tophus* was the highest. The *H. bacteriophora in vitro*-produced EPNs formulated in PPH had the highest areas under the survival curve of 1764. There was significantly lower survival of *in vivo*-produced IJs formulated in both Barricade^®^ gel and PPH for *S. innovationi* and *H. bacteriophora* while there was no difference in the survival of *S. tophus* IJs, produced *in vivo* and *in vitro*, for both formulations.

In the efficacy study (Table 2), there was no survival for the three control treatments after two weeks. However, for the EPNs produced *in vivo* and *in vitro*, and formulated in Barricade^®^ gel or PPH, the efficacy was 100% on Day 1. At six weeks, the efficacy of the different products declined in a range of 57–90% for both *in vivo* and *in vitro*-produced EPNs. At week eight, the *in vivo* and *in vitro*-produced EPNs in both the Barricade^®^ and PPH formulations were still effective in a range of 50–57% for *H. bacteriophora*. Both *Steinernema* species (*S. tophus* and *S. innovationi*) at eight weeks retained high levels of efficacy, with the *in vivo*-produced EPNs in Barricade^®^ gel and PPH, and the *in vitro*-produced EPNs in PPH causing mortalities of 90–93%, respectively (Table 2). With *S. innovationi* at eight weeks, efficacy was only retained in the *in vitro*-produced EPNs in PPH, causing a mortality of 93% of *T. molitor*. However, the *in vivo* and *in vitro*-produced EPNs in Barricade^®^ and *in vivo*-produced EPNs in PPH declined to 0% at eight weeks. There was a significant difference between the three isolates and the controls, formulations were compared within three species.

**Table 2:** Table showing infectivity of entomopathogenic nematodes produced *in vivo* and *in vitro* against *Tenebrio molitor* and formulated in either Barricade^®^ gel (B) or Potassium Polyacrylate Hydrogel (PPH) (LSD (*P* = 0,05) = 6,1742).

**Strain**	**Formulation**	**Day 1**	**2 weeks**	**4 weeks**	**6 weeks**	**8 weeks**
**SGI 245** *Heterorhabditis bacteriophora*	*In vivo* B	100 a	90 a	93 a	70 a	57 a
	*In vitro* B	100 a	83 a	77 b	70 a	50 a
	*In vivo* PPH	100 a	90 a	90 a	70 a	53 a
	*In vitro* PPH	100 a	90 a	80 b	73 a	53 a
	*In vivo* control	100 a	0 b	^*^	^*^	^*^
	*In vitro* control	100 a	0 b	^*^	^*^	^*^
**ROOI 352** *Steinernema tophus*	*In vivo* B	100 a	97 a	100 a	83 a	90 a
	*In vitro* B	100 a	97 a	73 c	67 b	^*^
	*In vivo* PPH	100 a	100 a	100 a	90 a	93 a
	*In vitro* PPH	100 a	100 a	80 b	67 b	93 a
	*In vivo* control	100 a	0 b	^*^	^*^	^*^
	*In vitro* control	100 a	0 b	^*^	^*^	^*^
**SGI 35** *Steinernema innovationi*	*In vivo* B	100 a	93 a	90 a	80 a	^*^
	*In vitro* B	100 a	80 b	77 a	57 b	^*^
	*In vivo* PPH	100 a	93 a	90 a	80 a	^*^
	*In vitro* PPH	100 a	80 b	80 a	60 b	93
	*In vivo* control	100 a	0 c	^*^	^*^	^*^
	*In vitro* control	100 a	0 c	^*^	^*^	^*^

* *there was no infective juvenile survival to conduct infectivity bioassay*.

**Figure 2:**
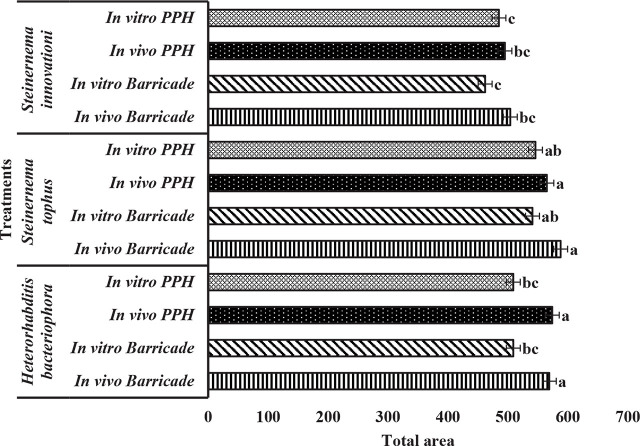
Area under the curve [analyzed using area under the disease progress curve (AUDPC) statistical analyses] for mealworm mortality following infection with three entomopathogenic nematode species, produced in vivo or in vitro and formulated in Barricade^®^ gel (B) or Potassium Polyacrylate Hydrogel (PPH), assessed at two weeks intervals over a period of eight weeks. Different letters at each bar indicate significant differences between treatments (α = 0.05), compared for each nematode species separately.

In Fig. 2, the Barricade^®^ gel formulation with *in vivo*-produced EPNs for the three isolates, *H. bacteriophora, S. tophus,* and *S. innovationi* had areas under the mortality curve of 569, 588, and 504, respectively. With *in vitro*-produced EPNs formulated in Barricade^®^ gel, the areas for *H. bacteriophora*, *S. tophus,* and *S. innovationi* were 509, 541, and 462, respectively. With the PPH formulation of *in vivo*-produced EPNs the areas under the mortality curve for the three isolates *H. bacteriophora, S. tophus,* and *S. innovationi* were 574, 565 and 495, respectively. With the *in vitro*-produced EPNs formulated in PPH, *H. bacteriophora*, *S. tophus* and *S. innovationi* had areas under the mortality curve of 509, 546, and 485, respectively. *In vivo H. bacteriophora* was better than *in vitro*, however there were no significant differences.

## Discussion

Gel formulations enhanced the survival of IJs, and the surviving IJs after storage were viable and able to cause mortality in *T. molitor*. In contrast, after two weeks, all the unprotected control non-formulated EPNs were dead while more than 50% of the formulated EPNs survived. Available data shows that EPNs can survive for more than three months when stored in water at 4 °C and 10 °C (Grewal, 2000); however, in this study the EPNs were subjected to extreme environmental stress as they were in a paste form (very high concentration). This confirmed that both formulations enhanced the survival of the three EPN isolates, produced both *in vivo* and *in vitro*. Musca et al. (2016) conducted a study to investigate if different *Steinernema carpocapsae* Weiser 1955 (Rhabditida, Steinernematidae) formulations using a low gel concentration of Barricade^®^ gel will protect EPNs from direct UV light (sunlight), if the other protective ingredients added to the gel would increase efficacy, and the survival of EPNs applied to foliage with the gel. They concluded that the gel, at low concentrations, protects EPNs, and addition of titanium dioxide enhanced the protective properties of the formulation. These results were in agreement with the current study. At eight weeks, the survival of EPNs, with both the Barricade^®^ gel and PPH formulations, were below 15% for *H. bacteriophora* (SGI 245) and *S. tophus* (ROOI 352), and 0% for *S. innovationi* (SGI 35). However, at 6 weeks, the survival levels ranged from 37–69% for all three EPNs and both formulations. The low level of survival observed in the controls (without gel) was likely due to the high concentration of IJs (in a paste form) used in the study, which was much higher than what is normally used in laboratory storage (Hazir et al., 2022), but more akin to commercial-level storage concentrations. In prior studies, gel formulations were demonstrated to protect EPNs from UV and/or desiccation in field applications and thereby increase efficacy (Shapiro-Ilan et al., 2010; 2016; Dito et al., 2016; Acar et al. 2022). In our study we have demonstrated that these gels can also be used for EPN formulation and enhance storage capacity.

Our findings are in line with other studies that indicated that similar gel formulations improve EPN storage capacity. For example, the results are in agreement with a study conducted by Abate et al. (2019), where it was concluded that nematode survival was highest in a gel medium, followed by a gel-soil mixture, and lowest in soil alone when stored for a period of 12 days. Leite et al. (2018) also assessed the performance of seven different formulation substrates with various combinations to preserve *Steinernema feltiae* Filipjev (Rhabditida, Steinernematidae) at three different temperatures: 15 °C, 25 °C, and 35 °C. The survival and mobility of *S. feltiae* IJs was recorded in mushroom compost, peat (sphagnum peat moss), potting mix, polyacrylamide gel, diatomaceous earth, vermiculite, and water as a control. It was concluded that the polyacrylamide gel formulation held higher numbers of nematodes in the substrate compared with the solid substrates. The mixture of vermiculite + double polyacrylamide gel provided the best preservation of IJs at 35 °C compared to the substrates tested separately. The combination of vermiculite with polyacrylamide gel preserved *S. feltiae* IJs with viability higher than 80% for at least 30 days at 35 °C, for 233 days at 25 °C, and 241 days at 15 °C.

Barricade^®^ gel is a superabsorbent polymer that is mixed with water to provide heat and dehydration protection. Potassium polyacrylate hydrogel has high water absorbance and retention properties. It quickly absorbs and holds a significant amount of water. It improves soil quality, increases seedling development, can release water slowly for crop uptake and it is nonpolluting (Costa et al., 2021). The two gels offer two vital properties, water retention and heat protection, that enhance the survival of EPNs.

*Heterorhabditis bacteriophora* had better IJ survival at eight weeks than both *Steinernema* species (*S. tophus* and *S. innovationi*). Entomopathogenic nematodes were able to survive up to week eight (56 days). Glazer et al. (2008) evaluated field efficacy of EPNs against the beetle *Maladera matrida* Brenske (Coleoptera: Scarabaeidae). They also found that EPNs could still be effective after 78 days in storage. Shapiro-Ilan et al. (2006) also conducted a study where they proved that nematode persistence can vary under differing soil biotic and abiotic conditions.

The use of the area under the curve for mortality of *T. molitor* infected with three EPNs produced *in vivo* or *in vitro* and with two formulations calculated from the accumulated mortality data over eight weeks provided very similar data, since a single IJ is capable of infecting and killing a host (Alonso et al., 2018). *Heterorhabditis bacteriophora* performed well, especially the two *in vivo* formulations. *Steinernema innovationi* was the worst performing isolate. The *in vivo*-produced IJs were less effective than the *in vitro* production method for all three EPN strains except for *S. tophus* Barricade^®^ gel formulations (Fig. 1).

The two *Steinernema* spp. were more virulent than the *Heterorhabditis* sp. eight weeks post storage (Table 2). These results contradict those reported by Bhairavi et al. (2021) where *H. bacteriophora* was more virulent than *Steinernema aciari* (Nematoda: Steinernematidae) Qiu et al. (2005) against *Odontotermes obesus* (Rambur) (Isoptera: Termitidae) and *Agrotis ipsilon* (Hufnagel) (Lepidoptera: Noctuidae). The discrepancy between studies may be due to strain differences or experimental conditions.

The *in vivo* technique is known to produce better quality IJs, however, these were less effective than *in vitro*-produced IJs. Furthermore, the *in vitro* method is a scalable technology that allows for the large-scale production of EPNs, while the *in vivo* production method can only be utilized for niche markets (McMullen et al., 2014). However, there are several approaches to increase *in vivo* production efficiency for EPNs (Shapiro-Ilan et al., 2022). In any case, our results indicated that the gels tested improve storage capacity for EPNs regardless of the production method.

Infective juveniles undergo a process called activation, where they infect the host, release the symbiotic bacteria, secrete toxin products, and undergo morphological change. Alonso et al. (2018) conducted a study in host-specific activation of EPN IJs where he concluded that the activation had a context-dependent influence on virulence and could be predictive of virulence in some cases, such as when IJ activation was especially low. This may explain the high increase in infectivity in week eight with both of the *Steinernema* spp.

There were no significant differences between the two formulations, Barricade^®^ gel and PPH, for similar strains and production methods. However, the PPH formulation is more accessible and cheaper than Barricade^®^ gel. Hence, the EPN product with the most commercial potential for improved storage would be the EPN strain ROOI 352 produced *in vitro* and formulated in PPH. Nevertheless, the two formulations also need to be compared for potential to enhance field efficacy in above- and belowground applications.

## Conclusion

Entomopathogenic nematodes were able to survive up to six weeks in both the Barricade^®^ gel and PPH formulations. In contrast, 100% of the control treatments died within two weeks. The *in vivo* production method was slightly less effective than the *in vitro* production method for all three EPN strains, in support of the *in vitro* production, which is a more commercially viable production method. The storage capacity of the two gel formulations were equally effective. This favors PPH formulations for storage capacity as PPH is readily available and is a less expensive option than Barricade^®^ gel. On that note, both commercial and small-scale farmers will be in a position to afford PPH-formulated EPN products. Moreover, PPH has a number of advantages over Barricade^®^ gel as it is considered environmentally friendly and has the ability to save water, fertilizer, and manpower; it improves nutrient usage; and it improves soil conditions for plants. Overall, the best commercial product would be the EPN strain *S. tophus* produced *in vitro* and formulated in PPH. However, both formulations still need to be evaluated for their potential to improve efficacy under field conditions.

## References

[j_jofnem-2025-0014_ref_001] Abate B. A., Slippers B., Wingfield M. J., Conlong D. E., Burger D. A., Hurley B. P. (2019). Virulence and survival of native entomopathogenic nematodes for the management of white grubs in South Africa. Journal of Biological Control.

[j_jofnem-2025-0014_ref_002] Acar I., Sipes B. (2022). Enhancing the biological control potential of *Steinernema feltiae* with protection from desiccation and UV radiation. Journal of Biological Control.

[j_jofnem-2025-0014_ref_003] Alonso V., Nasrolahi S., Dillman A. R. (2018). Host–specific activation of entomopathogenic nematode infective juveniles. Insects.

[j_jofnem-2025-0014_ref_004] Andalo V., Cavalcanti R.S., Molina J.P., Moino A. (2010). Substrates for storing entomopathogenic nematodes (Rhabditida: Steinernematidae, Heterorhabditidae). Scientia Agricola.

[j_jofnem-2025-0014_ref_005] Bhairavi K. S., Bhattacharyya B., Devi G., Bhagawati S., Das P. P. G., Devi E. B., Manpoong N. S. (2021). Evaluation of two native entomopathogenic nematodes against *Odontotermes obesus* (Rambur)(Isoptera: Termitidae) and *Agrotis ipsilon* (Hufnagel)(Lepidoptera: Noctuidae). Egyptian Journal of Biological Pest Control.

[j_jofnem-2025-0014_ref_006] Costa M. C. G., Freire A. G., Lourenço D. V., Sousa R. R. D., Feitosa J. P. D. A., Mota J. C. A. (2021). Hydrogel composed of potassium acrylate, acrylamide, and mineral as soil conditioner under saline conditions. Scientia Agricola.

[j_jofnem-2025-0014_ref_007] Dhiman J., Prasher S. O., El Sayed E., Patel R., Nzediegwu C., Mawof A. (2020). Use of polyacrylamide superabsorbent polymers and plantain peel biochar to reduce heavy metal mobility and uptake by wastewater–irrigated potato plants. Transactions of the ASABE.

[j_jofnem-2025-0014_ref_008] Dito D. F., Shapiro–Ilan D. I., Dunlap C. A., Behle R. W., Lewis E. E. (2016). Enhanced biological control potential of the entomopathogenic nematode, *Steinernema carpocapsae*, applied with a protective gel formulation. Biocontrol Science and Technology.

[j_jofnem-2025-0014_ref_009] Fisher B. A. (1970). Decision emergence: Phases in group decision–making. Communications Monographs.

[j_jofnem-2025-0014_ref_010] Glazer I., Goldberg A. (2008). Field efficacy of entomopathogenic nematodes against the beetle *Maladera matrida* (Coleoptera:Scarabaeidae). Bioco ntrol Science and Technology.

[j_jofnem-2025-0014_ref_011] Grewal P. S. (2000). Anhydrobiotic potential and long-term storage of entomopathogenic nematodes (Rhabditida: Steinernematidae). International Journal for Parasitology.

[j_jofnem-2025-0014_ref_012] Hatting J., Stock S. P., Hazir S. (2009). Diversity and distribution of entomopathogenic nematodes (Steinernematidae, Heterorhabditidae) in South Africa. Journal of Invertebrate Pathology.

[j_jofnem-2025-0014_ref_013] Hazir S., Kaya H., Touray M., Cimen H., Shapiro–Ilan D. S. (2022). Basic laboratory and field manual for conducting research with the entomopathogenic nematodes, Steinernema and *Heterorhabditis,* and their bacterial symbionts. Turkish Journal of Zoology.

[j_jofnem-2025-0014_ref_014] Jeger M. J., Viljanen–Rollinson S. L. H. (2001). The use of the area under the disease–progress curve (AUDPC) to assess quantitative disease resistance in crop cultivars. Theoretical and Applied Genetics.

[j_jofnem-2025-0014_ref_015] Leite L. G., Shapiro–Ilan D. I., Hazir S. (2018). Survival of *Steinernema feltiae* in different formulation substrates: Improved longevity in a mixture of gel and vermiculite. Biological Control.

[j_jofnem-2025-0014_ref_016] McMullen J. G., Stock S. P. (2014). *In vivo* and *in vitro* rearing of entomopathogenic nematodes (Steinernematidae and Heterorhabditidae). Journal of Visualized Experiments.

[j_jofnem-2025-0014_ref_017] Nouh G. M. (2021). Efficacy of the entomopathogenic nematode isolates against *Spodoptera littoralis* (Boisduval) and *Agrotis ipsilon* (Hufnagel) (Lepidoptera: Noctuidae). Egyptian Journal of Biological Pest Control.

[j_jofnem-2025-0014_ref_018] Ramakrishnan J., Salame L., Nasser A., Glazer I., Ment D. (2022). Survival and efficacy of entomopathogenic nematodes on exposed surfaces. Scientific Reports Journal.

[j_jofnem-2025-0014_ref_019] Ramakuwela T., Hatting H., Laing. M.D., Hazir S., Thiebaut N. (2016). *In vitro* solid-state production of *Steinernema innovationi* with cost analysis. Biocontrol Science and Technology.

[j_jofnem-2025-0014_ref_020] Ramakuwela T., Hatting J., Laing M. D., Thiebaut N., Hazir S. (2018). Biological characterization of the entomopathogenic nematode, *Steinernema innovationi*: a South African isolate. Journal of Nematology.

[j_jofnem-2025-0014_ref_021] Shapiro–Ilan D. I., Cottrell T. E., Mizell R. F., Horton D. L., Behle R. W., Dunlap C. A. (2010). Efficacy of *Steinernema carpocapsae* for control of the lesser peachtree borer, *Synanthedon pictipes*: Improved aboveground suppression with a novel gel application. Biological Control.

[j_jofnem-2025-0014_ref_022] Shapiro–Ilan D. I., Cottrell T. E., Mizell R. F., Horton D. L. (2016). Efficacy of *Steinernema carpocapsae* plus fire gel applied as a single spray for control of the lesser peachtree borer, *Synanthedon pictipes*. Biological Control.

[j_jofnem-2025-0014_ref_023] Shapiro–Ilan D. I., Lewis E. E. (2024). Entomopathogenic nematodes as biological control agents.

[j_jofnem-2025-0014_ref_024] Shapiro–Ilan D. I., Goolsby J. A. (2021). Evaluation of Barricade^®^ to enhance survival of entomopathogenic nematodes on cowhide. Journal of Invertebrate Pathology.

[j_jofnem-2025-0014_ref_025] Shapiro–Ilan D. I., Hazir S., Glazer I. (2022). Entomopathogenic nematodes as models for inundative biological control. Nematodes as Model Organisms.

[j_jofnem-2025-0014_ref_026] Shapiro–Ilan D. I., Stuart R. J., McCoy C.W. (2006). A comparison of entomopathogenic nematode longevity in soil under laboratory conditions. Journal of Nematology.

[j_jofnem-2025-0014_ref_027] Sharmila R., Priya M. S., Subramanian S., Poornima K., Pandiyan M. (2018). Review on ecology of entomopathogenic nematodes. Journal of Entomology and Zoology Studies.

[j_jofnem-2025-0014_ref_028] Somvanshi V. S., Koltai H., Glazer I. (2008). Expression of different desiccation–tolerance related genes in various species of entomopathogenic nematodes. Molecular and Biochemical Parasitology.

[j_jofnem-2025-0014_ref_029] Sunmola K. (2015). Transgenerational effects of aflatoxin B1 on the nematode *Caenorhabditis elegans*.

[j_jofnem-2025-0014_ref_030] Tarasco E., Fanelli E., Salvemini C., El-Khoury Y., Troccoli A., Vovlas A., De Luca F. (2023). Entomopathogenic nematodes and their symbiotic bacteria: from genes to field uses. Frontiers in Insect Science.

[j_jofnem-2025-0014_ref_031] Vashisth S., Chandel Y. S., Sharma P. K. (2013). Entomopathogenic nematodes–A review. Agricultural Reviews.

[j_jofnem-2025-0014_ref_032] Yooyangket T., Muangpat P., Polseela R., Tandhavanant S., Thanwisai A., Vitta A. (2018). Identification of entomopathogenic nematodes and symbiotic bacteria from Nam Nao National Park in Thailand and larvicidal activity of symbiotic bacteria against *Aedes aegypti* and *Aedes albopictus*. PlOS One.

